# Error in Figure

**DOI:** 10.1001/jamahealthforum.2023.0812

**Published:** 2023-04-21

In the Research Letter titled “Care Experiences and Visit Use of Physician and Nonphysician Medicare Patients,”^1^ published online March 10, 2023, in part B of the Figure, the label to the left of 0 in the forest plot should be “Fewer visits” and the label to the right of 0 should be “More visits.” This article has been corrected.
